# The importance of Doppler ultrasound in pediatric inflammatory bowel disease

**DOI:** 10.1590/0100-3984.2023.56.5e3-en

**Published:** 2023

**Authors:** Dolores Bustelo, Tatiana Fazecas

**Affiliations:** 1 Radiologist at the Clínica CETAC - Diagnóstico por Imagem, Curitiba, PR, Brazil. Email: doloresbustelo@gmail.com; 2 Head of the Department of Imaging of the Hospital Municipal Jesus, Pediatric Radiologist at Dasa, Rio de Janeiro, RJ, Brazil. Email: tatifazecas@hotmail.com

Ultrasound is one of the most widely used imaging methods in the evaluation of abdominal diseases because it is noninvasive, affordable, and widely available. The main advantage of ultrasound is that it does not use ionizing radiation, a feature that is essential for the evaluation of pediatric patients.

Inflammatory bowel diseases (IBDs) comprise a group of diseases that cause chronic inflammation of the intestine, including Crohn’s disease, ulcerative colitis, and inflammatory bowel disease unclassified. The incidence and prevalence of IBDs have increased over the last 50 years, with an incidence of 25% in the pediatric population, being more aggressive in this age group^(^1^)^.

In making a diagnosis of pediatric IBD, it can be a challenging to choose the most informative diagnostic tests and to classify its different subtypes correctly. Ileocolonoscopy, upper gastrointestinal endoscopy, and magnetic resonance enterography, as well as, in some cases, capsule endoscopy, are first-line methods employed in making the initial diagnosis, allowing the location and degree of intestinal inflammation to be evaluated. Reevaluation during treatment is costly and often requires another sedation procedure, which can cause stress and anxiety in children. Noninvasive monitoring is increasingly used to verify the resolution of intestinal inflammation after clinical remission has been achieved, particularly in the pediatric population^(^2^)^.

Various studies have shown that ultrasound accurately detects, localizes, and characterizes inflammation of the intestinal wall, as well as allowing the evaluation of extraintestinal changes, with a good negative predictive value for IBD^(^3^)^. It is possible to evaluate IBD activity by using color Doppler ultrasound, which underscores the relevance of the method and provides important data for the monitoring and treatment of pediatric IBD.

In addition to the data obtained with color Doppler, ultrasound provides other important information in the evaluation of patients with IBD, with emphasis on the determination of the wall thickness in the bowel loops, the presence of regional lymph nodes, hypertrophy of the adipose tissue of the mesentery and the presence of free fluid in the peritoneal cavity^(^1^)^, as well as complications arising from the underlying disease.

Contrast-enhanced ultrasound can be used to accurately identify inflamed bowel loops. For a long time, its use was limited to the adult population because of safety concerns. However, there is evidence, albeit limited, that it is safe for use in children, although evidence of its usefulness in evaluating IBD in pediatric patients is still scarce. The advantage of contrast-enhanced ultrasound in the pediatric population remains to be proven; because the method requires intravenous access, its acceptability for use in children continues to be a concern^(^4^)^.

An article published in this issue of Radiologia Brasileira^(^5^)^ masterfully demonstrates the objective assessment of vascular flow in the bowel walls, through determination of the number of Doppler signals/cm^2^, transforming that assessment into numerical data. That means that the examination can be performed by different doctors, with reproducibility, making it possible to compare results between observers and between time points, thus reducing subjectivity and increasing accuracy. The article also demonstrated that the correlation between color Doppler ultrasound of the intestinal wall and the fecal calprotectin level shows high sensitivity and specificity for identifying inflammatory activity in pediatric IBD, corroborating data in the literature^(^1^)^.

Ultrasound has been widely used to evaluate intestinal loops in pediatric patients, from the neonatal period to adolescence. It is an excellent diagnostic imaging method that does not employ ionizing radiation, and it can be indicated as an initial examination in the investigation of suspected IBD in pediatric patients, although is mainly useful in monitoring such patients, providing important data on the evolution of the disease, and evaluating the response to treatment^(^6^)^. The scientific article in focus^(^5^)^ is extremely relevant and demonstrates the importance of ultrasound for the diagnosis of and evaluation of the treatment response in IBD.
